# Medications Used for Cognitive Enhancement in Patients With Schizophrenia, Bipolar Disorder, Alzheimer’s Disease, and Parkinson’s Disease

**DOI:** 10.3389/fpsyt.2018.00091

**Published:** 2018-04-04

**Authors:** Wen-Yu Hsu, Hsien-Yuan Lane, Chieh-Hsin Lin

**Affiliations:** ^1^Graduate Institute of Clinical Medical Science, China Medical University, Taichung, Taiwan; ^2^Changhua Christian Hospital, Changhua, Taiwan; ^3^School of Medicine, Chung Shan Medical University, Taichung, Taiwan; ^4^Psychiatry, China Medical University and Hospital, Taichung, Taiwan; ^5^Psychiatry, Kaohsiung Chang Gung Memorial Hospital, Kaohsiung, Taiwan

**Keywords:** cognitive impairment, medication, schizophrenia, bipolar disorder, Alzheimer’s disease, Parkinson’s disease

## Abstract

**Background/aims:**

Cognitive impairment, which frequently occurs in patients with schizophrenia, bipolar disorder, Alzheimer’s disease, and Parkinson’s disease, has a significant impact on the daily lives of both patients and their family. Furthermore, since the medications used for cognitive enhancement have limited efficacy, the issue of cognitive enhancement still remains a clinically unsolved challenge.

**Sampling and methods:**

We reviewed the clinical studies (published between 2007 and 2017) that focused on the efficacy of medications used for enhancing cognition in patients with schizophrenia, bipolar disorder, Alzheimer’s disease, and Parkinson’s disease.

**Results:**

Acetylcholinesterase inhibitors and memantine are the standard treatments for Alzheimer’s disease and Parkinson’s disease. Some studies have reported selective cognitive improvement in patients with schizophrenia following galantamine treatment. Newer antipsychotics, including paliperidone, lurasidone, aripiprazole, ziprasidone, and BL-1020, have also been reported to exert cognitive benefits in patients with schizophrenia. Dopaminergic medications were found to improve language function in patients with Parkinson’s disease. However, no beneficial effects on cognitive function were observed with dopamine agonists in patients with schizophrenia. The efficacies of nicotine and its receptor modulators in cognitive improvement remain controversial, with the majority of studies showing that varenicline significantly improved the cognitive function in schizophrenic patients. Several studies have reported that *N*-methyl-d-aspartate glutamate receptor (NMDAR) enhancers improved the cognitive function in patients with chronic schizophrenia. NMDAR enhancers might also have cognitive benefits in patients with Alzheimer’s disease or Parkinson’s disease. Raloxifene, a selective estrogen receptor modulator, has also been demonstrated to have beneficial effects on attention, processing speed, and memory in female patients with schizophrenia.

**Conclusion:**

Clinical trials with larger sample sizes evaluating comprehensive cognitive domains are warranted to examine the efficacy of medications in cognitive enhancement in patients with schizophrenia, bipolar disorder, Alzheimer’s disease, and Parkinson’s disease.

## Introduction

Cognitive function, including neurocognition and social cognition, is associated with mental processes that lead to the acquisition of information and knowledge. It drives an individual’s understanding and actions in his or her environment. Neurocognition and social cognition can predict the functional outcome in an individual with schizophrenia or bipolar disorder (1). Normal cognitive changes generally occur with aging, such as the decline in processing speed, memory, language, and visuospatial and executive function abilities (2). Therefore, elderly people require longer time to learn and recall information (3).

Cognitive impairment is the frequent symptom occurring in nonelderly patients with schizophrenia or other neurodegenerative disorders (4). Cognitive dysfunction in patients with schizophrenia was described by Kraepelin more than a century ago (5). Increased awareness and advancements in the area of neuropsychological assessment and neuroimaging techniques have now rendered cognitive impairment an important focus of theories on the etiology and treatment of schizophrenia.

A large-scale comprehensive quantitative meta-analysis study reported that patients with schizophrenia have moderately to severely impaired neurocognition, particularly in terms of global verbal memory functioning (6). Furthermore, multiple analyses in the Clinical Antipsychotic Trials of Intervention Effectiveness study on schizophrenia have suggested that patients with schizophrenia are characterized by a broad cognitive deficit (7). Another meta-analysis study reported that deficits across multiple social cognitive domains in patients with schizophrenia were clear and replicated, particularly in the domains of theory of mind (ToM) and emotion perception (8). Patients with schizophrenia are impaired in various cognitive functions, including both neurocognition and social cognition, which are associated with a functional outcome. Neurocognitive function was also found to be impaired in euthymic patients with bipolar disorder (9). In addition, a meta-analysis of patients with bipolar disorder in the euthymic stage reported the presence of deficits in emotion processing and ToM (10). Patients with bipolar disorder are also impaired in both neurocognition and social cognition. Moreover, neurocognitive impairment has been found to be similar among patients with bipolar disorder and patients with schizophrenia (11).

Mild cognitive impairment (MCI) and dementia are not a part of the normal aging process. MCI has been defined as a greater decline in cognition without significant daily life interference than that in normal aging considering the education and the age of an individual (12). In individuals with dementia, these symptoms involve mental decline that is sufficiently severe to disrupt their daily life activities (13).

The mechanisms of cognitive impairment are different among patients with schizophrenia, bipolar disorder, Alzheimer’s dementia, and Parkinson’s disease. Schizophrenia is a complex disorder. Cognitive deficit has been considered as one of the core symptoms of schizophrenia (14–16). The deficiency in proactive control is directly related to the impairment in the dorsolateral prefrontal cortex (DLPFC). This impairment might be related to DLPFC dysfunction; impaired DLPFC connectivity with the striatum, the thalamus, and the parietal cortex; and alterations in the levels of neurotransmitters, including glutamate, γ-aminobutyric acid (GABA), and dopamine (17). The mechanism of cognitive impairment in patients with bipolar disorder has not yet been clearly elucidated. Some researchers believe that cognitive decline is linked with the mechanisms of neuroinflammation and neuroprotection in bipolar disorder (18). Alzheimer’s disease, a neurodegenerative disease with a progressive course, is characterized by two specific lesions, extracellular β-amyloid plaques and neurofibrillary tangles (19–21). The glutamate system has also been reported to play a crucial role in cognitive function (22). Synaptic dysfunction in Alzheimer’s disease has been presumed to be related to glutamate receptors. Synaptic transmission and synaptic plasticity can be damaged by the β-amyloid protein. Metabotropic and *N*-methyl-d-aspartate glutamate receptors (NMDARs) have also been reported to be involved in Alzheimer’s disease (23). Cognitive deterioration in patients with Parkinson’s disease occurs due to the dysmetabolism of both amyloid protein and α-synuclein and cholinergic dysfunction (24, 25).

As mentioned earlier, although the mechanisms of cognitive impairment vary among different neurodegenerative disorders, cognitive impairment has been currently regarded as an important determinant of functional domains and is a potential treatment goal in patients with schizophrenia, bipolar disorder, Alzheimer’s disease, and Parkinson’s disease.

Cognitive enhancement still remains a clinically unresolved challenge. Till date, there is no effective treatment available for enhancing cognitive function in patients with schizophrenia, bipolar disorder, Alzheimer’s disease, and Parkinson’s disease. This review article systemically examines and presents an update on pharmacological cognitive enhancement in patients with schizophrenia, bipolar disorder, Alzheimer’s disease, and Parkinson’s disease.

## Method of Review

We performed a search of studies related to our topic using PubMed Clinical Queries in July 2017. We searched for human clinical trials focusing on cognitive enhancement in patients with schizophrenia, bipolar disorder, Alzheimer’s disease, and Parkinson’s disease, excluding other cognitive disorders. The following search string was used: “(Cognitive function OR Cognitive enhancer OR Cognition improvement) AND medication AND (Schizophrenia OR Bipolar disorder OR Alzheimer disease OR Parkinson’s disease).” We limited the search results to articles published between January 1, 2007, and June 30, 2017, and those with “clinical trial” as the article type, which resulted in a total of 799 articles. We excluded the string “Alzheimer’s disease OR dementia” from our search due to the availability of several lines of evidence indicating treatment efficacy and US Food and Drug Administration (FDA) approval (26–28). Several compounds for enhancing cognition in patients with schizophrenia and degenerative diseases were reported in the retrieved articles. We review and summarize these clinical trials in the present article.

## Acetylcholinesterase Inhibitors

Three acetylcholinesterase inhibitors, donepezil, galantamine, and rivastigmine, have been approved by the US FDA for treating Alzheimer’s disease (29–31). Treatment with acetylcholinesterase inhibitors resulted in higher concentrations of acetylcholine, leading to the inhibition of aggregation of β-amyloid and increased communication between neurons, which in turn decreases cognitive decline (13).

A total of 11 clinical trials pertained to treatment with acetylcholinesterase inhibitors for improving cognitive function in patients with schizophrenia, which included five studies on galantamine, four studies on donepezil, and one study each on rivastigmine and neuromidin (32–42). All the five small-sample, randomized, double-blind, placebo-controlled studies on galantamine adjunctive treatment reported no beneficial effects on cognitive function in the galantamine-treated patients with schizophrenia (32–36). Among the four clinical studies on donepezil adjunctive treatment, three randomized, double-blind, placebo-controlled trials reported no significant improvement in neurocognitive function in the donepezil-treated patients with schizophrenia (37–39). The other open-label trial showed that donepezil adjunctive treatment in stable schizophrenic patients resulted in significant improvement in mental set-shifting ability (*p* < 0.05), long-term memory and learning ability (*p* < 0.05), and attention (*p* < 0.05) among 13 patients (40). The randomized crossover design study on rivastigmine adjunctive therapy reported no significant cognitive improvement in the rivastigmine-treated patients (41). Finally, the randomized controlled study on neuromidin, a nonselective acetylcholinesterase inhibitor, evaluated 55 marked neurocognitive deficits in patients with schizophrenia and demonstrated positive improvement in visuospatial memory, attention, retention and retrieval of data, and planning (42). Thus, the majority of these clinical studies have demonstrated no significant improvement in neurocognitive function in patients with schizophrenia treated with acetylcholinesterase inhibitors.

Three clinical trials pertained to treatment with acetylcholinesterase inhibitors for enhancing cognition in patients with bipolar I disorder (43–45). Iosifescu et al. (43) administered galantamine ER 8–24 mg daily to patients with bipolar disorder for 4 months in an open-label study. After treatment, these patients showed obvious improvement in verbal episodic memory (*p* < 0.05), attention (*p* < 0.05), and subjective cognitive scores (*p* < 0.01) (43). Gildengers et al. (44) conducted a small-scale, 12-week, open-label pilot study in elderly patients with bipolar I or II disorder. They observed that acute treatment with donepezil (5–10 mg/day) was not related to amelioration of cognition and daily life activities. Ghaemi et al. (45) performed a double-blind, placebo-controlled study to investigate the cognition efficacy of galantamine augmentation in patients with euthymic bipolar disorder. They reported improvement in California Verbal Learning Test (CVLT) Total Learning in the galantamine group from baseline and improvement in the category fluency and the Delis–Kaplan executive function system trail-making conditions in the placebo group from baseline. There was no significant difference between galantamine and placebo groups possibly due to small sample size. Hence, the efficacy of acetylcholinesterase inhibitors on cognition in patients with bipolar disorder remains uncertain due to the concerns of placebo effect, limited sample size, and the inconsistent results of previous studies. Further trials with larger sample sizes addressing the concern of placebo effect are warranted.

Five clinical studies evaluated the efficacies of acetylcholinesterase inhibitors on cognition in patients with Parkinson’s disease, which included two clinical trials on galantamine and two on rivastigmine.

A small-sample, open-label, controlled trial on patients with Parkinson’s dementia who received galantamine demonstrated higher scores on the Mini-Mental State Examination (MMSE), Alzheimer’s Disease Assessment Scale-Cognitive Subscale (ADAS-cog), frontal assessment battery (FAB), and clock drawing test than those in the control group at the end of the study (46). Another double-blind, placebo-controlled study did not find improvement in visuospatial performance, memory, or attention/execution in patients who received galantamine treatment among those with nondemented Parkinson’s disease (47). Both clinical trials demonstrate the lack of evidence to confirm the cognitive benefits of galantamine in patients with Parkinson’s disease.

A small-sample, 24-week, randomized, double-blind, placebo-controlled, crossover, single-site study of MCI in patients with Parkinson’s disease demonstrated a significant effect of rivastigmine transdermal patch in terms of the Everyday Cognition Battery Memory Test (*p* < 0.05); in contrast, no treatment effect was found in terms of Dementia Rating Scale-2, Montreal Cognitive Assessment (MoCA), and NeuroTrax Computerized Cognitive Battery scores (48). Another 12-month observation study involving patients with Parkinson’s disease having cognitive dysfunction reported that patients treated with rivastigmine showed significantly greater improvement in MoCA scores (*p* < 0.01) than that of the controls (49). Both clinical trials have demonstrated the evidence of cognitive benefits in patients with Parkinson’s disease and cognitive impairment treated with rivastigmine.

Thus, the majority of clinical studies have demonstrated significant efficacy of acetylcholinesterase inhibitor, rivastigmine, in Parkinson’s disease patients with MCI or dementia. These trials did not show effectiveness of acetylcholinesterase inhibitor on cognition in patients with Parkinson’s disease. To gain generalizability, further large-scale double blinded trials are warranted.

## Memantine

Memantine, an antagonist of NMDARs, has been approved by the US FDA for treating moderate-to-severe Alzheimer’s disease (50). It has been reported that memantine could provide both neuroprotection and symptomatic improvement through rapid, moderate-affinity, voltage-dependent NMDAR channel blockade (51). It also has favorable effects on cognitive impairment in other neurodegenerative diseases. We identified seven clinical studies that evaluated the efficacy of memantine treatment on cognitive function in schizophrenic patients in our search. In three of these studies, a 6-week, open-label study; an 8-week, double-blind, placebo-controlled study; and a 12-week, placebo-controlled study, memantine (adjunctive therapy) showed no efficacy on cognition improvement (52–54). However, in the other 12-week, small-sample with 21 patients, double-blind, placebo-controlled study, patients with refractory schizophrenia who were treated with memantine showed a significantly greater MMSE improvement (*p* < 0.01) than the improvement of those who received placebo (55). A 26-week, randomized, double-blind, placebo-controlled crossover study also demonstrated the efficacy of memantine on cognitive function, with a composite memory score comprising verbal recognition memory and paired associates learning task scores on the Cambridge Neuropsychological Test Automated Battery (effect size = 0.30, *p* < 0.05), in 52 clozapine-refractory schizophrenic patients (56). However, the continued open-label 1-year extension study did not show the cognitive benefit at weeks 26 and 52 (57). Another 12-week, randomized, double-blind, placebo-controlled clinical trial also reported significant improvement in the MMSE score in the memantine intervention group at week 6 (*p* < 0.05) and week 12 (*p* < 0.01) in patients with schizophrenia treated with risperidone (58). Although some of the aforementioned studies have reported positive effects of memantine intervention on cognitive function, the results were inconsistent possibly due to the variations in treatment duration. Thus, the effect of memantine on cognitive impairment in patients with schizophrenia remains uncertain. Further double-blind, placebo-controlled trials with longer treatment duration are needed to determine the efficacy of memantine on cognitive impairment in patients with schizophrenia.

We did not find any clinical trial on the effect of memantine on cognitive enhancement in patients with bipolar I disorder in our search. However, we identified one clinical study evaluating the effect of memantine on cognition in patients with Parkinson’s disease complicated by dementia. This small-sample, long-term, open-label, controlled trial showed that patients with Parkinson’s disease who received memantine exhibited significant improvement in the MMSE (*p* < 0.05), ADAS-cog (*p* < 0.05), clock drawing test (*p* < 0.05), and FAB scores (*p* < 0.01), compared to that in the controls at week 24 (59). At the end of 52 weeks, significant changes were observed in the 12-item Neuropsychiatric Inventory scale scores compared to those at baseline in the memantine-treated patients (*p* < 0.05) (59). This clinical trial showed that memantine appeared to be effective on cognition. The effects of memantine on cognition in Parkinson’s disease have not yet been established because of the open-label study design and small sample size. Hence, further well-designed, double-blind, placebo-controlled trials with larger sample sizes are required to determine the efficacy of memantine on cognitive impairment in patients with Parkinson’s disease.

## Antipsychotics

Antipsychotics are the major medications for schizophrenia or psychosis for controlling psychotic symptoms. In addition to psychotic symptoms, extensive evidence indicates the presence of cognitive impairment accompanying schizophrenia. The cognitive efficacy of antipsychotics has gained more research attention in the recent 10 years. Several clinical trials on second-generation antipsychotics have focused on cognition in patients with schizophrenia.

In a 24-week, non-randomized, open-label trial, the mean change in Wisconsin Card Sorting Test Keio Version in the number of categories achieved (*p* < 0.05) and the perseverative errors in Nelson (*p* < 0.05) from baseline at the second stage was found to be significantly greater in the risperidone long-acting injection (RLAI) group than that in haloperidol decanoate depot group (60). The mean changes from baseline in the individual St. Marianna University School of Medicine’s Computerized Memory Test, including immediate verbal recall (*p* < 0.01), delayed verbal recall (*p* < 0.01), delayed verbal recognition (*p* < 0.01), memory scanning test (*p* < 0.05), and memory filtering test (*p* < 0.05), were found to be significantly greater in the group switched to RLAI than those in haloperidol decanoate depot group (60). In a 6-month, open-label, randomized, controlled study, 30 patients with schizophrenia who were treated with RLAI were randomly allocated to the RLAI-continued group or the paliperidone palmitate long-acting injection (PP) group. The Brief Assessment of Cognition in Schizophrenia score (BACS) assessing the attention and processing speed item showed greater improvement in the PP group than that in the RLAI group (*p* < 0.05) (61). Another 12-week, small-sample, open-label study reported that the mean change from baseline in the z-score of the digit sequencing task was significantly improved after switching from risperidone to paliperidone in elderly schizophrenic patients (62). Furthermore, a 12-week, randomized, open-label study on schizophrenic patients demonstrated significantly greater improvements in recall after an interference phase in the verbal learning test in the paliperidone-switch group than those in the risperidone-continuation group, but not in the other six neurocognitive domains measured (63). A 6-week, placebo- and active-controlled study followed by a 6-month double-blind extension trial indicated that lurasidone 160 mg daily was superior to both placebo and quetiapine in terms of the neurocognitive composite at week 6 (*p* < 0.05), whereas there was no difference among lurasidone 80 mg daily, quetiapine XR 600 mg daily, and placebo. In the double-blind extension study, the lurasidone (40–160 mg daily) group showed significantly better cognitive performance than that in the quetiapine XR (200–800 mg daily) group at 6 months (*p* < 0.01) (64). Patients who received final doses of lurasidone 120 and 160 mg daily showed significantly greater improvement in the overall cognitive performance compared to that with quetiapine XR at 6 months, while 40 and 80 mg daily treatment showed a trend toward significance at 6 months (65). A 16-week, randomized, double-blind, placebo-controlled trial reported that adding ziprasidone to clozapine in patients with schizophrenia significantly improved semantic fluency (*p* < 0.01) (66). Another 18-week, randomized, double-blind trial that compared clozapine and ziprasidone showed improvement in the composite cognitive score from baseline in both groups, although the improvements were significantly greater in the ziprasidone-treated group (*p* < 0.05) (67). A prospective, 12-week, multicenter, noncomparative, open-label study of aripiprazole in schizophrenic patients showed a significant improvement in verbal cognition from week 4 in terms of the long-term free recall in the CVLT over the scheduled visits in the trial (*p* < 0.01) and a significant improvement in phonemic (letter) subtest of the verbal fluency test from baseline to week 12 (*p* < 0.05) (68).

BL-1020, a new GABA-enhanced antipsychotic compound, 20–30 mg daily demonstrated significantly greater improvements in cognitive functioning as measured by the BACS composite score when compared to those with placebo (*p* < 0.01), risperidone 2–8 mg daily (*p* < 0.05), and BL-1020 10 mg daily (*p* < 0.05) after 6 weeks in a 6-week, randomized, double-blind, controlled trial (69). These clinical trials support the importance of cognitive improvement in patients with schizophrenia as a new focus of antipsychotic treatment. To summarize the efficacy on cognition, RLAI may be more effective than haloperidol decanoate depot, PP may be more effective than RLAI, lurasidone may be more effective than quetiapine XR, ziprasidone may be more effective than clozapine, and BL-1020 seems to be more effective than placebo from current clinical trials.

We did not find any clinical trial on antipsychotics for improving cognition in patients with bipolar disorder, Alzheimer’s disease, or Parkinson’s disease.

## Dopamine Agonists and Agents for Enhancing Dopamine Activity

Dopamine, a brain catecholamine originating from subcortical neurons, has been reported to supplement the activity of several neural circuitries belonging to both subcortical and neocortical structures (70). However, the dopamine hypothesis of schizophrenia suggests that dopamine agonist medication supposedly worsens the positive symptoms of schizophrenia (71). The D2-selective blockade by antipsychotics has provided strong support for the dopamine hypothesis. However, the roles of other dopamine receptors in schizophrenia remain unclear. D1 and D2 receptors have been reported to exert opposing actions on intracellular signaling molecules and often have different physiological effects (72). It is well known that the D1 receptors of the prefrontal cortex are involved in working memory (73). D3 receptors are predominantly found in the limbic regions that modulate memory, emotions, and motivation (74). The D3 receptors are presumed to be associated with cognitive functioning. In our search, we identified only few articles pertaining to the effect of dopamine agonists on cognitive enhancement in schizophrenia. In a randomized controlled trial, pramipexole, a dopamine D3 agonist, was added for up to 12 weeks to ongoing antipsychotic treatment (75). The trial found no differences in cognition between the pramipexole and the placebo groups (75). In another randomized controlled trial of DAR-0100A, a dopamine-1 receptor agonist, 3 weeks of intermittent treatment with 0.5 or 15 mg or placebo showed no significant treatment effects on working memory domains of the Measurement and Treatment Research to Improve Cognition in Schizophrenia (MATRICS) (76). Thus, there is a lack of evidence to support the efficacy of dopamine agonists or agents for cognitive enhancement in patients with schizophrenia.

Regarding bipolar disorder, we found only one clinical trial on a dopamine agonist that increases dopamine activity for cognition enhancement. An 8-week, double-blind, placebo-controlled trial involving 35 euthymic patients subgroup with bipolar disorder demonstrated a significant improvement by treatment with pramipexole, an agonist for the D2, D3, and D4 dopamine receptors, in terms of the WAIS Digits Backward (*p* < 0.05) and Stroop Color Word tests (*p* < 0.05) (77). Due to the small sample and the short duration in this study, the evidence of dopamine agonists for cognitive enhancement in patients with bipolar disorder is limited.

However, we did not find any study on dopamine agonists for cognitive enhancement in patients with dementia in our search. Dopamine agonists represent a valid therapeutic option in Parkinson’s disease. However, the efficacy of dopamine agonists on cognitive function in Parkinson’s disease has not been well studied. In a randomized crossover study, rotigotine, cabergoline, nor levodopa improved cognition in 40 patients with early, mild Parkinson’s disease compared to that in the off-treatment group (78). An open-label study reported that cognitive improvements as assessed by the MoCA-Japanese version (MoCA-J) total score and the subscore of delayed recall were found with 4–7 months of dopaminergic medication (l-dopa, a dopamine agonist, selegiline) treatment among 27 drug-naive patients with Parkinson’s disease (79). Thus, the effect of dopamine agonists on cognition in Parkinson’s disease remains uncertain due to the limitation in study design (only one randomized crossover study and one open-label study) and the small sample size.

Due to the limited clinical trials on the medications of dopamine activity enhancement found in our search, further well-designed clinical trials on dopamine-related medications, especially D1 and D3 agonists, for cognitive enhancement are still needed.

## Nicotine and its Receptor Modulators

A large body of evidence derived from studies supports the notion that nicotine has cognitive-enhancing effects. Several clinical studies have investigated the effects of nicotine and relative medications on cognitive function in patients with schizophrenia. In a randomized, placebo-controlled, crossover design study, 28 schizophrenic and 32 healthy nonsmokers received transdermal nicotine (14 mg/24 h) or a placebo patch (80). It was observed that nicotine had beneficial effects on attention in both schizophrenic and healthy nonsmokers, with intermediate performance by *ad libitum* smoking (80). Nicotine was related to a greater improvement in the inhibition of impulsive responses in patients with schizophrenia 3 h after each patch application (80). Another double-blind, randomized, placebo-controlled, crossover, 3-day pilot trial investigated the efficacy of intravenous nicotine on symptomatology and cognition in schizophrenic patients and reported no significant dose × time effects on the Stroop Color-Word Test and continuous performance task (81). The efficacy of nicotine on cognition in patients with schizophrenia is still controversial based on both the aforementioned studies. Further well-designed, larger sample size, longer duration, double-blind, placebo-controlled trials are required to determine the efficacy of nicotine on cognition in patients with schizophrenia.

Varenicline, a partial agonist at the α4β2 receptor and also a full agonist at the α7 nicotine acetylcholine receptor, demonstrated significant improvement in several cognition domains related to verbal learning and memory, but not in domains related to attention or visuospatial learning or memory in a 6- to 9-week open-label study (82). However, a randomized, double-blind, placebo-controlled, 8-week study reported that varenicline showed significant improvement in nonperseverative errors in the Digital Symbol Substitution Test (*p* < 0.05) and the Wisconsin Card Sorting Test (*p* < 0.05) in subjects with schizophrenia (83). Varenicline was found to significantly reduce the Stroop Interference (*p* < 0.01) and the Continuous Performance Test hit reaction time (*p* < 0.01) compared to that with placebo among smokers but not among nonsmokers (83). In a phase 2, multicenter, double-blind, randomized, placebo-controlled trial involving patients with stable schizophrenia, AZD3480, another selective agonist of α4β2 and α2β2 nicotinic receptors, failed to improve cognition relative to placebo (84). These studies have thus reported the efficacy of varenicline on cognition improvement in patients with schizophrenia.

Encenicline is a novel selective α7 nicotinic acetylcholine receptor agonist. In a phase 2, 12-week, double-blind, randomized, placebo-controlled, parallel-design study, schizophrenic patients were randomized to receive either encenicline 0.27 or 0.9 mg or placebo daily (85). Patients who received 0.27 mg encenicline daily showed better Overall Cognition Index from the CogState computerized battery (*p* < 0.05) than that of patients who received placebo (85). Patients who received 0.9 mg encenicline daily showed greater improvement in Schizophrenia Cognition Rating Scale (*p* < 0.05) and in the Positive and Negative Syndrome Scale cognition domain (*p* < 0.01) compared to those in patients who received placebo (85). Another 12-week, randomized exploratory trial of an α7 nicotinic receptor agonist (TC-5619) demonstrated that TC-5619 led to significant improvement in Groton Maze Learning Task (GMLT; executive function) of the CogState Schizophrenia Battery (*p* < 0.05) at week 4 in patients with schizophrenia, but not at weeks 8 and 12 (86). However, TC-5619 led to significant improvement in GMLT at weeks 4 and 12 in the tobacco-use subgroup as well (86). A yet another phase 2, 24-week, randomized, double-blind study on patients with schizophrenia demonstrated that TC-5619 did not support a benefit for cognitive impairment (87). RG3487, an α7 nicotinic acetylcholine receptor partial agonist was reported to show no improvement in cognitive impairment in an 8-week, double-blind, randomized study on patients with schizophrenia (88). Thus, only encenicline demonstrated cognitive benefits in patients with schizophrenia patients in these clinical trials on α7 nicotinic receptor agonists.

We did not identify any clinical study evaluating the efficacy of nicotine and relative medications on cognition in patients with bipolar disorder in our search; however, we found two studies evaluating the effect of nicotine or relative medication in patients with Alzheimer’s disease and Parkinson’s disease. One was a phase 2, 12-week, double-blind, placebo-controlled study that reported that neither AZD3480 nor donepezil was significantly superior to placebo in terms of ADAS-Cog in patients with mild to moderate Alzheimer’s disease (89). The other was a double-blind, placebo-controlled, crossover design study in which acute transdermal nicotine patches (7 mg for 24 h) were found to improve impaired controlled semantic processing (*p* < 0.001) in patients with Parkinson’s disease (90). Nevertheless, due to the small sample size and the short duration of these trials, further well-designed trials with a larger sample size and a longer duration are needed to determine the efficacy of nicotine on cognition in patients with Parkinson’s disease.

## *N*-Methyl-d-Aspartate Receptor Enhancers

*N*-methyl-d-aspartate glutamate receptors play an important role in learning and memory *via* neural plasticity, including long-term depression and potentiation. We identified five clinical studies evaluating the cognitive efficacy of NMDAR enhancers, including d-cycloserine, d-serine, sodium benzoate, sildenafil, and l-carnosine, in patients with schizophrenia in our search.

A 16-week, randomized, double-blind, double-dummy, parallel trial of adjuvant d-cycloserine, glycine, or placebo reported that glycine or d-cycloserine was not better than placebo in terms of cognitive performance (91). In another randomized, double-blind, parallel-group, 8-week trial, add-on d-cycloserine 50 mg administered once-weekly showed no improvement in cognitive performance compared to that with placebo (92). In a 4-week, open-label study, there was no obvious change in MATRICS for a d-serine dose of 30 mg/kg; however, improvement with a large effect size in MATRICS was observed for d-serine 60 mg/kg (*p* < 0.01) or more, suggesting a dose-dependent effect (93). d-Serine doses of 60 mg/kg or more led to a significant change across all domains, except working memory (93).

d-Amino acid oxidase (DAAO), a flavoenzyme of peroxisomes, can metabolize d-serine and d-alanine and exists in the central nervous system. Sodium benzoate, a DAAO inhibitor, enhances NMDAR function by inhibiting DAAO activity and increases the levels of d-amino acids. In a randomized, double-blind, placebo-controlled, 6-week study of 1 g daily sodium benzoate or placebo as an adjunctive to antipsychotics in patients with stabilized chronic schizophrenia, the benzoate group showed better performance than the placebo group in terms of processing speed (*p* < 0.05) and visual learning and memory (*p* < 0.05) (94).

Regarding sildenafil, a phosphodiesterase-5 inhibitor, Goff et al. (95) reported that it could increase the concentrations of cyclic guanosine monophosphate operated by NMDARs intracellularly, which has been presumed to be linked to memory consolidation and long-term potentiation. They administered a single oral dosage of sildenafil 50 or 100 mg or placebo in a randomized manner during a 48-h interval between prescriptions to 17 adult outpatients with schizophrenia who were under stable antipsychotic treatment; however, neither dosage of sildenafil showed any significant impact on cognitive performance compared to that with placebo.

l-Carnosine, an antioxidant and antiglycation agent, has been shown to exert protective effects on cultured neurons against the deprivation of oxygen and glucose and NMDA-related neurotoxicity. Furthermore, studies have reported that it can reduce glutamate excitotoxicity effect in wild-type and histidine-decarboxylase-knockout mice (96, 97). In a 3-month, double-blind study, 75 symptomatically stable patients with chronic schizophrenia were assigned to receive either adjuvant l-carnosine 2 g daily or placebo randomly (98). Patients who received l-carnosine showed better performance in nonreversal set-shifting test than that of patients who received placebo; however, there was no significant difference in reversal reaction times and errors between the two groups (98). In addition, patients who received l-carnosine displayed better strategic efficiency (*p* < 0.05) with less perseverative errors (*p* < 0.05) than that of the control group (98).

Therefore, our search for NMDAR enhancers suggests that d-serine, sodium benzoate, and l-carnosine showed possible benefits on cognition in patients with schiozophrenia.

Regarding the enhancing effect of NMDARs on cognition in patients with Alzheimer’s disease, a randomized, double-blind, placebo-controlled, 24-week trial demonstrated that patients with early-phase Alzheimer’s disease who received sodium benzoate exhibited a greater improvement in ADAS-cog (*p* = 0.0021, 16 weeks; *p* = 0.0116, 24 weeks; *p* = 0.0031, end point) and additional cognitive score (*p* = 0.007 at end point) compared to the improvement of those patients who received placebo (99).

We identified another clinical study that evaluated the efficacy of NMDAR enhancers on cognitive function in patients with Parkinson’s disease. This 8-week, double-blind, placebo-controlled trial showed that sarcosine, an NMDAR coagonist, did not show significant improvement in Cognitive Abilities Screening Instrument and MMSE scores compared to placebo (100).

However, we did not find any clinical trial on the efficacies of NMDAR enhancers on cognitive function in patients with bipolar disorder in our search.

## Erythropoietin (EPO)

Erythropoietin regulates red blood cell production or erythropoiesis. EPO improves oxygen capacity in the blood by boosting red blood cell production. Several animal model and cell studies have demonstrated that EPO exhibits a neuroprotective effect through antioxidant, antiapoptotic, anti-inflammatory, neurotrophic, angiogenic, and synaptogenic activities (101–103). In our search, we identified four clinical trials that evaluated the effect of EPO treatment on cognitive functions.

In a multicenter, randomized, double-blind, placebo-controlled, 12-week, phase II trial, patients with schizophrenia who received 40,000 IU recombinant human EPO for 3 months with a weekly short (15 min) intravenous infusion were found to experience a significant benefit compared to that of controls in cognitive test package, including Repeatable Battery for the Assessment of Neuropsychological Status subtests (delayed memory, language-semantic fluency, attention) and Wisconsin Card Sorting Test-perseverative errors (*p* = 0.010) (104). A three-phase exploratory study involving 10 patients with Parkinson’s disease receiving recombinant human EPO treatment reported that all the patients showed a favorable and significant increase in the total Dementia Rating Scale score over their baseline status (*p* < 0.01), with the attention subtest also contributing to this change (105). Another study reported that recombinant human EPO administration significantly improved the attention/memory domain score of the Non-Motor Symptoms Scale for Parkinson’s disease (*p* < 0.01) and the cognitive domain score of the 39-item Parkinson’s Disease Questionnaire (*p* < 0.01) at 12 months in 26 patients with Parkinson’s disease (106). In another double-blind, randomized, placebo-controlled, phase II trial on bipolar disorder, EPO was shown to improve processing speed for learning, attention (*p* < 0.05), executive functions (*p* < 0.05), recognition of happy faces (*p* < 0.05), and sustained attention (*p* < 0.05) (107). However, owing to the small sample size or the short duration of these trials, further well-designed trials with a larger sample size and a longer duration are warranted to determine the efficacy of EPO on cognition in these diseases.

## Selective Estrogen Receptor Modulator

Estrogen receptors are widely distributed over the brain, particularly in the amygdala and the hippocampus that are associated with memory and learning. Some studies investigating the effects of estrogen on postmenopausal women have provided evidence for the efficacy of sex hormones on cognition (108, 109). In our search, we identified four clinical studies that had evaluated the efficacy of raloxifene, a selective estrogen receptor modulator, in patients with schizophrenia. In a double-blind, randomized, placebo-controlled, parallel-design, 12-week study involving postmenopausal women with schizophrenia, significant differences were found in the executive (phonemic fluency task, *p* < 0.05) and memory domains (learning curve, *p* < 0.05) in 16 patients receiving 60-mg daily raloxifene treatment (110). This study demonstrated improvement in some cognitive domains following raloxifene treatment compared to that with placebo in postmenopausal female patients with schizophrenia. However, the sample size of this study is too small to demonstrate generalizability of this result.

Another study identified in our search evaluated the efficacy of raloxifene in patients with Alzheimer’s disease. In that randomized, double-blind, placebo-controlled, 12-month pilot study among women with late-onset Alzheimer’s disease, it was observed that patients who had received raloxifene (120 mg/day) and placebo showed no significant differences in terms of ADAS-cog change scores at 12 months (111). However, our search yielded no clinical study in the recent decade that had investigated the efficacy of selective estrogen receptor modulators on cognitive enhancement in patients with bipolar disorder and Parkinson’s disease. Further clinical studies are required to determine the efficacy of raloxifene on cognitive enhancement in these female patients.

## Dehydroepiandrosterone (DHEA)

Dehydroepiandrosterone, an important corticosteroid, is a precursor for not only androgenic but also estrogenic steroids. DHEA and its sulfated form (DHEAS) have been reported to modulate the functioning of neurons (112). We found one clinical trial that investigated the efficacy of DHEA on cognition in patients with schizophrenia. It was a 12-week, randomized, double-blind, placebo-controlled study that demonstrated no improvement in cognitive performance (most notably memory) following treatment with DHEA (113). However, in our search, we did not find any clinical study in the recent decade that had evaluated the efficacy of DHEA on cognition in patients with bipolar disorder, Alzheimer’s disease, or Parkinson’s disease.

## Conclusion

Schizophrenia, bipolar disorder, Alzheimer’s disease, and Parkinson’s disease are devastating brain disorders that are associated with lifetime disability and dysfunction in society. Improvement in cognitive function is critical for patients with these disorders. The acetylcholinesterase inhibitors donepezil and rivastigmine might have beneficial effects on cognitive deficits in patients with Parkinson’s disease. The effects of memantine on cognition in patients with schizophrenia and Parkinson’s disease still remain undetermined. Newer antipsychotics, including paliperidone, lurasidone, aripiprazole, ziprasidone, and BL-1020, have shown possible cognitive benefits than other antipsychotics or placebo in patients with schizophrenia in some clinical trials. Varenicline showed efficacy in terms of cognition improvement in schizophrenic patients, whereas nicotine did not. The α7 nicotinic receptor agonist, such as encenicline, might have cognitive benefits in patients with schizophrenia. Dopamine agonists or agents for enhancing dopamine activity showed little effect on cognitive improvement in patients with bipolar disorder or Parkinson’s disease. The NMDAR enhancers, including d-serine, sodium benzoate, and l-carnosine showed cognitive benefits in patients with schizophrenia. NMDAR enhancers might also have cognitive benefits in patients with Alzheimer’s disease or Parkinson’s disease. EPO demonstrated little evidence of cognitive benefits among patients with schizophrenia, bipolar disorder, or Parkinson’s disease. The selective estrogen receptor modulator raloxifene might have cognitive benefits in postmenopausal female patients with schizophrenia. Although few multiyear, prospective, clinical studies evaluating cognitive enhancement following treatment have been conducted, the results of several compounds for the diseases described in this review remain inconsistent. Therefore, additional long-term, well-designed, and large-scale trials are warranted to determine the effects of these medications on cognition improvement in patients with schizophrenia, bipolar disorder, Alzheimer’s disease, or Parkinson’s disease.

## Author Contributions

W-YH, H-YL, and C-HL involved in conception, literature review and interpretation, and manuscript writing. All authors reviewed the manuscript.

## Conflict of Interest Statement

The authors declare that the research was conducted in the absence of any commercial or financial relationships that could be construed as a potential conflict of interest. The reviewer TI and handling Editor declared their shared affiliation.

## Abbreviations

CATIE, clinical antipsychotic trials of intervention effectiveness; ToM, theory of mind; MCI, mild cognitive impairment; DLPFC, dorsolateral prefrontal cortex; GABA, glutamate, γ-aminobutyric acid; NMDARs, *N*-methyl-d-aspartate glutamate receptors; FDA, Food and Drug Administration; ADAS-cog, Alzheimer’s Disease Assessment Scale-Cognitive Subscale; MMSE, Mini-Mental State Examination; FAB, frontal assessment battery; MoCA, Montreal Cognitive Assessment; RLAI, risperidone long-acting injection; BACS, Brief Assessment of Cognition in Schizophrenia score; PP, paliperidone palmitate long-acting injection; CVLT, California Verbal Learning Test; MATRICS, measurement and treatment research to improve cognition in schizophrenia; MoCA-J, MoCA-Japanese version; GMLT, Groton Maze Learning Task; DAAO, d-amino acid oxidase; EPO, erythropoietin; DHEA, dehydroepiandrosterone; DHEAS, DHEA sulfated form.
